# Attitudes and Practices of Complementary and Alternative Medicine Among Adolescents in Saudi Arabia

**DOI:** 10.5539/gjhs.v7n1p173

**Published:** 2014-08-22

**Authors:** Abdulrahman O. Musaiger, Nada A. Abahussain

**Affiliations:** 1 Arab Center for Nutrition, Bahrain; 2 Directorate of School Health, Ministry of Education, Al-Khobar, Saudi Arabia

**Keywords:** adolescents, alternative medicine, complementary medicine, Saudi Arabia, traditional medicine

## Abstract

The aim of this study was to investigate the attitudes and use of complementary and alternative medicine (CAM) among Saudi Arabian adolescents. A multistage stratified sampling method was used to select 736 adolescents (358 males, 378 females) aged 15–19 years from secondary schools. The study was carried out in Al-Khobar city, Eastern region of Saudi Arabia. The findings revealed that the use of CAM by adolescents in their lifetime ranged from 1.6% for acupuncture to 58.6% for honey treatment, with significant differences between genders, except in the use of dietary supplements, black cumin, and acupuncture therapies. Females were more likely to use CAM for treating abdominal pains, cold and flu, and cough than males (P < 0.000). Family members and friends (67.7%) were the main source of CAM usage, followed by television (10%), and Internet (8%). Religious and medicinal herb healers were the CAM healers most commonly visited by adolescents. Nearly 21–43% of adolescents had positive attitudes toward CAM, with some significant differences between males and females. It can be concluded that CAM is widely used by Saudi adolescents, but caution should be exercised for the safe usage of some CAM treatments. CAM should not be ignored; however there is an urgent need to establish regulations for CAM usage.

## 1. Introduction

Complementary and alternative medicine (CAM) could be defined as “a group of diverse medical and health care systems, practices and products that are not presently considered to be part of conventional Western medicine” (NIH, 2014). The use of CAM for treatment of several illnesses has increased markedly, both in developing and developed countries. It has been estimated that the use of CAM or traditional medicine ranges from 31% in Belgium to 92% in Ethiopia (WHO, 2002).

The use of CAM in the pediatric population has risen significantly during the past decade (Tsao & Zeltzer, 2005; Barnes et al., 2007). In the USA, 79% of adolescents aged 14–19 years have used CAM during their lifetime (Wilson et al., 2006). In the UK, 41% of children and adolescent patients had used at least one type of CAM in the past year of the survey (Crawford et al., 2006). Parents’ reasons for using CAM for their children included efficacy of some CAM treatments, fear of adverse drug effects, dissatisfaction with conventional treatment, and the need for personal attention (Kemper et al., 2008). However, the use of CAM is higher among children and adolescents with chronic diseases, such as those with cancer, diabetes, sickle cell, cystic fibrosis, asthma, and eating disorders (Crawford et al., 2006; Barnes et al., 2007; Sibinga et al., 2006). Herbal and home therapies were also commonly used in the pediatric emergency population (Lanski et al., 2003).

Studies on the use of CAM in the Arab Gulf countries are very limited; almost all the studies are focused on adults. In Saudi Arabia, Al-Faris et al. (2008) found that 68% of adults used CAM during the past 12 months of the survey. Jan et al. (2009) reported that 52% of Saudi families used CAM routinely, based on their own strong personal beliefs. Another study among Saudi women showed that the application of CAM ranged from 10% for acupuncture to 81% for reading the holy Quran (spiritual healing) (Musaiger et al., 2012a). Since none of the previous studies targeted adolescents, we attempted to carry out this study to highlight the attitudes and practices of CAM among a representative sample of adolescents in Saudi Arabia.

## 2. Methods

### 2.1 Participants and Sampling

The target group of this study was adolescents aged 15–19 years living in Al-Khobar city, in the Eastern region of Saudi Arabia. A multistage stratified sampling method was used to collect the sample. First, Al-Khobar city was administratively divided into three geographical areas. Then, two secondary schools were selected from each geographical area (one for boys and another for girls), using simple random method. One class from each level (10–12 levels) was then selected from each school by simple random method. All the students who were available in the selected classes participated in the study. The total sample obtained was 736 (358 males and 378 females). Data were collected during the school year 2009–2010.

### 2.2 Ethical Permission

The ethical permission to carry out this study was obtained from the Department of Studies and Research at the Center of Planning and Development in General Directorate of School Education in Eastern Province, Saudi Arabia.

### 2.3 The Questionnaire

A pretested self-reported questionnaire was used to collect the data (Musaiger et al., 2012a). The questionnaire consisted of two parts: CAM practices and attitudes toward CAM. The attitudes were measured using a three-point Likert scale. The students completed the questionnaire in their classrooms under supervision of their teachers and research assistants. All the questionnaires were checked before the students left the classrooms.

### 2.4 Data Analysis

The data were analyzed using the Epi-info statistical package (CDC, 2012). A chi-square test was calculated to determine the significance of the association between genders and dependable variables.

## 3. Results

The CAM most commonly used by Saudi adolescents during their lifetime according to gender is shown in Table 1. Dietary supplements (59.4%), honey healing (58.6%), reading the holy Quran (47.3%), black cumin healing (40.4%), and medicinal herbs (37%) were the CAM commonly used. Except for black cumin healing, dietary supplements, and acupuncture, there were significant differences between genders in the use of CAM (p-value ranged from 0.023–0.000).

**Table 1:** CAM most commonly used by Saudi adolescents during their lifetime according to gender

CAM	Male (%)	Female (%)	P-value	Total (%)
Honey healing	45.2	62.7	0.019	58.6
Dietary supplements	57.8	60.8	0.404	59.4
Reading of Quran	37.2	56.9	0.000	47.3
Black cumin healing	41.6	39.2	0.496	40.4
Medicinal herbs	28.9	44.7	0.000	37.0
Cautery	10.1	5.6	0.023	7.7
Bloodletting (*Hijama*)	4.5	1.3	0.010	2.9
Acupuncture	1.7	1.6	0.924	1.6

The common health symptoms treated using CAM among Saudi adolescents according to gender are presented in Table 2. Abdominal pains (47%), cold and flu (37.6%), and cough (31.3%) were the main health symptoms treated by CAM. Significant differences were observed between males and females in the proportion of treating health symptoms, except for treatment of diarrhea, dental pain, and allergy.

**Table 2:** Common health symptoms treated by CAM among Saudi adolescents during their lifetime

Health symptoms	Male (%)	Female (%)	*P-value*	Total (%)
Abdominal pains	37.2	56.3	0.000	47.0
Cold and flu	31.0	43.9	0.000	37.6
Cough	25.4	36.8	0.000	31.3
Diarrhea	28.8	29.1	0.921	28.9
Dental pain	23.7	27.8	0.212	25.8
High body temperature	19.6	13.8	0.035	16.6
Headache	19.3	13.5	0.034	16.3
Allergy	15.9	11.2	0.056	13.5

The main single source of CAM information used by Saudi adolescents according to gender is shown in Table 3. Family members and friends were the major single source of CAM information (67.7%), followed by television (10.1%), and Internet (8.0%). Females were more likely to depend on family members and friends (70.1% vs. 65.1%) than males, while males were more likely to deped on television (13.1% vs. 7.1%) than females as the source of information on CAM. The association between source of CAM information and gender was statistically significant (p < 0.036).

**Table 3:** Main single source of information on CAM used by Saudi adolescents according to gender

Source	Male		Female		Total	
	No.	%	No.	%	No.	%
Family members and friends	233	65.1	265	70.1	498	67.7
Television	47	13.1	27	7.1	74	10.1
Internet	22	6.1	37	9.8	59	8.0
Newspapers and magazines	20	5.6	21	5.6	41	5.6
Books	12	3.4	12	3.2	24	3.3
Health practitioners	10	2.8	4	1.1	14	1.9
Various sources	14	3.9	12	3.2	26	3.5
Total	358	100.0	378	100.0	736	100.0

**Figure 1:**
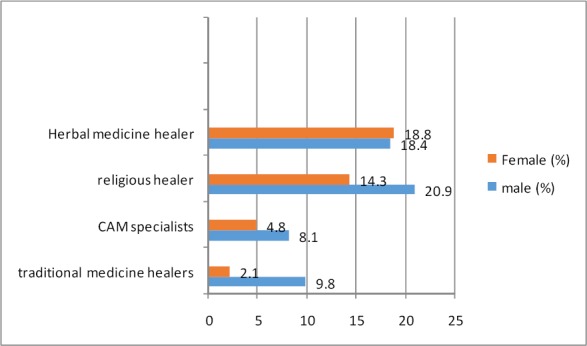
Proportion of Saudi adolescents who seek treatment by CAM healers during their lifetime (% of those who seek treatment)

The types of CAM providers visited by Saudi adolescents during their lifetime are illustrated in Figure 1. Herbal healers (18.6%) and religious healers (spiritual healers) were the CAM providers most visited by adolescents. Males were more likely to visit spiritual healers, CAM specialists, and traditional medicine healers than females.

The attitudes of Saudi adolescents toward CAM according to gender are given in Table 4. A relatively high percentage of adolescents believed that medicinal herbs are safe to use (34.6% males, 42.6% females) (p < 0.081). Nearly 30% of males and 21% of females believed that CAM is better than conventional medicine in treating diseases (p < 0.000). About one-fifth of adolescents agreed with the statement “vitamins and minerals can be used without prescription”.

**Table 4:** Attitudes of Saudi adolescents toward CAM according to gender

Statement	Gender	Agree %	Disagree %	Don’t know %	P-value
CAM is better for treatment of diseases than conventional medicine	Male	29.9	39.7	30.4	0.000
	Female	20.9	35.7	43.4	
CAM can be used without consulting a medical practitioner	Male	24.3	57.3	18.4	0.022
	Female	33.3	48.9	17.7	
Medicinal herbs are safe to use	Male	34.6	33.2	32.1	0.081
	Female	42.6	30.2	27.2	
Medicinal herbs can be used safely without prescribed medications	Male	27.7	33.2	39.1	0.024
	Female	20.4	41.5	38.1	
Vitamins and minerals can be used without prescription	Male	19.3	65.6	15.1	0.299
	Female	23.3	60.3	16.4	
Overdose of vitamins and minerals supplements do not cause any side effects	Male	8.7	64.5	26.8	0.624
	Female	7.8	67.9	24.4	

## 4. Discussion

This study indicates that CAM is commonly used by Saudi adolescents, and there were significant differences between males and females in some uses of CAM. Culturally-based CAM practices, such as spiritual healing, and use of honey and black cumin therapies, were commonly used compared with other CAM practices. Saudi adolescents used CAM for healing common health symptoms but not for chronic diseases. Family members and friends were the main single source of CAM information. Religious and herbal healers were the most visited by adolescents. Although a high proportion of the participants disagreed with the attitudes statements related to CAM usage, about one-fifth to one-third believe in the positive benefits of CAM.

The use of CAM by Saudi adolescents during their lifetime ranged from 1.6% for acupuncture to 58.6% for honey treatment. These figures are comparable with those reported by Jan et al. (2009) in Saudi Arabia, where 42% of families used CAM for their children. The common CAM practices used by Saudi adolescents were healing by honey, black cumin, reading the Quran, and medicinal herbs. These types of CAM were found to be commonly used by adults in Saudi Arabia (Al-Faris et al., 2008; Jan et al., 2009; Musaiger et al., 2012a). The use of these types of CAM are usually based on cultural and religious background, not only in Saudi Arabia, but in all Muslim communities, as there are several positive statements in the Quran and by the prophet Mohammed advocating the healing properties of these CAM practices (Musaiger et al., 2012b).

Spiritual therapy such as reading or hearing the voice of the Quran is commonly used by Muslim populations around the world for healing several chronic diseases and reducing stress. In many verses, the Quran focuses on the healing of disease (Sadeghi, 2011). Studies in Western countries have suggested that religious beliefs and practices may contribute to decreased stress and increased sense of well-being and enhanced immune system functioning (Kemper et al., 2008).

Dietary supplements, mainly vitamins and minerals, were commonly used by Saudi adolescents in this study (59.4%). This percentage is higher than the 32% reported earlier among Saudi female adolescents (Abahussain & Taha, 2007), but it is within the range reported by Dorsch and Bell (2005) in their review on use of dietary supplements by adolescents (12–19 years), where usage ranged from 10–74%. These reviewers found that the most commonly cited reasons for use of dietary supplements by adolescents were: to maintain or improve health, to build muscle, to increase energy, to decrease or increase body weight, to increase athletic performance, and to balance the diet. Medicinal herbs were widely used by Saudi female adolescents (44.7%) compared with their male counterparts (28.9%). This is probably due to the wide use of weight reduction herbs by females, as they are more concerned about their weight. It was found that 15% of females in this study used herbal tea to reduce weight compared to 4% of males (not shown in the Tables). However, further investigations are needed to confirm this conclusion.

Saudi adolescents used CAM for healing of common health symptoms. This was expected as most chronic diseases occur in adulthood. However, females were more likely to treat cough, cold and flu, and abdominal pains, than males, and the differences were statistically significant. This could be due to the fact that girls at this age (14–19) could be suffering menstrual pain, which is normally characterized as abdominal pain. The high use of CAM for healing cough, cold, and flu by females compared to males, is probably due to the higher prevalence of these health symptoms among females. It was reported that women were more likely to have flu symptoms than men. This is mainly due to the fact that women tend to spend more time with children at home, who are more likely to have flu-like illness in the first place (Flusurvey, 2014). This is probably true in this study, as girls in Arab Gulf countries, including Saudi Arabia, are more likely to stay at home than boys due to cultural restrictions on girls going outside the home (Musaiger et al., 2014). There is no clear explanation for significant use of some CAM practices between genders. This could be the subject of future research.

Family members and friends were the main source of information on CAM for Saudi adolescents. This creates the need to educate the public, especially parents and peers, on the advantages and disadvantages of CAM. Television (10.1%) was the second source of information on CAM. Abahussain and Taha (2007) reported that 60% of female Saudi adolescents depended on television as a source of self-medication, which indicates the role of television in educating the public. In general, adolescents seem to be more open than adults in using CAM therapies, and adolescents are more willing to use CAM if their parents also use these therapies (Kemper et al., 2008).

Nearly 20–40% of Saudi adolescents had positive attitudes toward CAM. Despite the growing popularity of CAM approaches for pediatric illness, questions remain regarding the efficacy of these interventions (Tsao & Zeltzer, 2005). Although CAM is perceived positively, several side-effects of this intervention have been reported, including death, anaphylaxis, renal failure, and malignancy. Adverse effects occur directly or from drug interaction (McCann & Newell, 2006). However, CAM cannot be ignored by health care providers. Therefore, caution and regulation may justify using this kind of therapy (Niggemann & Gruber, 2003).

This study is the first attempt to investigate the use of CAM among adolescents in the Arab Gulf countries, and indicates that CAM is widely practiced by Saudi adolescents. Health care providers need to be aware of the use of CAM by their patients and policy makers should consider the impact of the current lack of regulation of CAM, especially in pediatric practice (Wilson et al., 2006). Health education, through mass media, should focus on risk awareness and safety of CAM. Further studies on knowledge, attitudes, and practice of CAM among children and adolescents are needed to highlight the growing use of this type of healing and to help in establishing relevant regulation of CAM. We hope that this study stimulates other investigators in the Arab Gulf countries to carry out in-depth studies on this topic.

## References

[ref1] Al-Faris E. A, Al-Rowais N, Mohamed A. G, Al-Rukban M. O, Al-Kurdi A, Balla A, Sheikh A (2008). Prevalence and pattern of alternative medicine use:the results of a household survey. Ann Saudi Med.

[ref2] Abahussain N. A, Taha A. Z (2007). Knowledge and attitudes of female school students on medications in eastern Saudi Arabia. Saudi Med J.

[ref3] Barnes P. M, Bloom B, Nahin R. L (2008). Complementary and alternative medicine use among adults and children:United States. Natl Health Stat Report.

[ref4] Centers for Disease Control and Prevention. (CDC) (2012). Epi info.

[ref5] Crawford N. W, Cincotta D. R, Lim A, Powell C. V. E (2006). A cross-sectional survey of complementary and alternative medicine use by children and adolescents attending the university hospital of Wales. BMC Complement Altern Med.

[ref6] Dorsch K. D, Bell A (2005). Dietary supplement use in adolescents. Curr Opin Pediatr.

[ref7] FluSurvey (2014). Results of 2014 Survey.

[ref8] Jan M. M, Basamh M. S, Bahassan O. M, Jamal-Allail A. A (2009). The use of complementary and alternative therapies in Western Saudi Arabia. Saudi Med J.

[ref9] Kemper K. J, Vohra S, Walls R (2008). The use of complementary and alternative medicine in pediatrics. Pediatrics.

[ref10] Lanski S. L, Greenwald M, Perkins M, Simon H. K (2003). Herbal therapy use in a pediatric emergency department population:Expect the unexpected. Pediatrics.

[ref11] McCann L. J, Newell S. J (2006). Survey of pediatric complementary and alternative medicine use in health and chronic illness. Arch Dis Child.

[ref12] Musaiger A. O, Janahi M. H, Al-Dayeni A (2012a). Usage of alternative and traditional medicine among Saudi women. Arab J Food Nutr.

[ref13] Musaiger A. O, Janahi M. H, Najem F, Al-Saad N (2012b). Alternative medicine in Bahrain community. Arab J Food Nutr.

[ref14] Musaiger A. O, Al-Roomi K, Bader Z (2014). Social, dietary and lifestyle factors associated with obesity among Bahraini adolescents. Appetite.

[ref15] National Institute of Health (2014). National Center for Complementary and Alternative Medicine.

[ref16] Niggemann B, Gruber C (2003). Side-effects of complementary and alternative medicine. Allergy.

[ref17] Sadeghi H (2011). Voice of Quran and health:a review of performed studies in Iran. Qual Quran Med.

[ref18] Sibinga E. M, Shindell D. L, Casella J. F, Duggan A. K, Wilson M. H (2006). Pediatric patients with sickle cell disease:use of complementary and alternative therapies. J Altern Complement Med.

[ref19] Tsao J. C. I, Zeltzer L. K (2005). Complementary and alternative approaches for pediatric pain:a review of the state-of-the-science. eCAM.

[ref20] WHO (2002). Traditional medicine growing needs and potential. WHO Policy perspectives on Medicines, N0.002, May 2002.

[ref21] Wilson K. M, Klein J. D, Sesselberg T. S, Yussman S. M, Markow D. B, Green A. E, Gray N. J (2006). Use of complementary medicine and dietary supplements among U.S. adolescents. J Adolesc Health.

